# Blood Vessels as Regulators of Neural Stem Cell Properties

**DOI:** 10.3389/fnmol.2019.00085

**Published:** 2019-04-12

**Authors:** Andromachi Karakatsani, Bhavin Shah, Carmen Ruiz de Almodovar

**Affiliations:** ^1^European Center for Angioscience, Medicine Faculty Mannheim, Heidelberg University, Mannheim, Germany; ^2^Institute for Transfusion Medicine and Immunology, Medicine Faculty Mannheim, Heidelberg University, Mannheim, Germany

**Keywords:** blood vessels, development, adult neurogenesis, neurovascular, NPCs, NSCs, metabolic regulation

## Abstract

In the central nervous system (CNS), a precise communication between the vascular and neural compartments is essential for proper development and function. Recent studies demonstrate that certain neuronal populations secrete various molecular cues to regulate blood vessel growth and patterning in the spinal cord and brain during development. Interestingly, the vasculature is now emerging as a critical component that regulates stem cell niches during neocortical development, as well as during adulthood. In this review article, we will first provide an overview of blood vessel development and maintenance in embryonic and adult neurogenic niches. We will also summarize the current understanding of how blood vessel-derived signals influence the behavior of neural stem cells (NSCs) during early development as well as in adulthood, with a focus on their metabolism.

## Concomitant Development of the Neural and Vascular Compartments of the CNS

**Figure 1:**
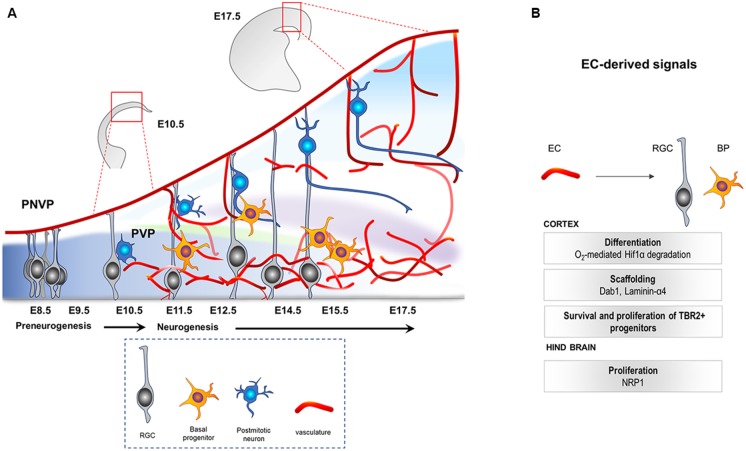
**(A)** Illustration of the developing mouse neocortex. At E8.5 till E10.5 the tissue is hypoxic, harboring mainly the apical progenitors (RGCs, gray) that come from neuroepithelial cells. PNVP is already established during this time. PVP ingression occurs, decreasing the hypoxia, around E11.5 onwards and is followed by generation of other cell types like Tbr2+ BP (yellow) and neurons (blue) by asymmetric divisions of the RGCs. PNVP, perineural vascular plexus; PVP, periventricular plexus; RGC, radial glial cells; BP, basal progenitors; EC, Endothelial cell. **(B)** Vascular-derived cues that mediate survival, growth and proliferation in the developing central nervous system (CNS).

In a murine model, the central nervous system (CNS) starts to develop at around E7.5–E8 when the neural plate forms the neural tube. At the rostro-caudal axis, the neural tube starts to partition into the rostral vesicle consisting of the forebrain, midbrain and hindbrain, while the caudal vesicle develops into the spinal cord. This is followed by extensive levels of progenitor proliferation, differentiation and generation of neurons that migrate to their specific regions where they connect and form synapses. The mammalian neocortex (forebrain-derived telencephalic region) is defined by six layers of neurons generated from a single layer of proliferating progenitor cells (neuroepithelial cells) that become highly polarized at their apicobasal axis (Breunig et al., 2011). This progenitor population, called radial glial cells (RGCs, also known as the primary neural stem cells (NSCs) of the CNS; Rakic, 2009; Rash et al., 2018), initially (E10.5–E12.5) undergoes extensive symmetric divisions to expand, followed by differentiation into neurons or basal progenitors (BPs; Tbr2+, E12.5 onwards; Paridaen and Huttner, 2014; Figure 1A). They later undergo final symmetric divisions to also generate pyramidal neurons (Martínez-Cerdeño et al., 2006). Once generated, postmitotic neurons use glia (RGC)-dependent mode of migration, followed by glia-independent mode of migration, to finally attain their positions in the cortex (Nadarajah et al., 2001).

Recent studies have shown that the CNS is vascularized at the same time as the formation of the neural compartment. Two independent vascular plexuses, the perineural plexus (PNVP/pial vessels) and the periventricular plexus (PVP), contribute to CNS vascularization. Between E8.5 and E10, the CNS starts to get vascularized by the PNVP that arises from the mesoderm-derived angioblasts (Hogan et al., 2004; Engelhardt and Liebner, 2014). This plexus covers the entire CNS by E9, however, it seems to lack any spatial or temporal gradient during its development (Vasudevan et al., 2008). While the PNVP is already ensheathing the CNS by E9, vessel ingression from the PVP into the cortex occurs 2 days later, at around E11.5 (Vasudevan et al., 2008). The PVP arises from the basal vessel located in the basal ganglia primordium coming from the cervical pharyngeal arch arteries (Hiruma et al., 2002). In contrast to vessel sprouting from the PNVP, PVP sprouts invade the neocortex from E11 onwards displaying a developmental gradient instructed by specific cues from the ventral and dorsal homeobox transcription factors (Vasudevan et al., 2008). Sprouts from the pial vessels (PNVP) surrounding the neocortex also invade the cortex radially by E12.5, a time point when PVP-derived sprouts have already entered and started to branch in the intermediate area (subventricular zone, SVZ) of the cortex. Subsequently, within the neural parenchyma, sprouts from the PNVP and the PVP branch and fuse to build the vascular network of the developing cortex (Figure 1A; Vasudevan et al., 2008).

## Association of the Vasculature With NSCs/NPCs During Development

During growth and regeneration, stem cells from different tissues (e.g., pancreas, liver, adipose tissue and CNS) grow in close proximity to blood vessels, which supply oxygen and nutrients to meet the high metabolic demands of the stem cells (Rafii et al., 2016). In addition, apart from acting as conducts for oxygen and nutrients, blood vessel-derived molecules can regulate stem cell properties. It is well described that a direct association of endothelial cells (ECs) and hematopoietic stem cells (HSCs) regulate the self-renewal and differentiation of HSCs *via* angiocrine derived signals (Rafii et al., 2016). HSCs and embryonic NSCs share similar molecular and genetic profile (Ivanova et al., 2002), suggesting that their response to different angiocrine cues may also be common. While the angiocrine potential of ECs to modulate NSCs is well described in adult neurogenesis (see below), much less is known during development. Below, we describe the few studies addressing this topic in the developing mouse brain.

### Association of Embryonic NSCs and Vessels During Development

Although less characterized than adult NSCs, multiple *in vitro* studies have determined the association of embryonic NSCs and vasculature. ECs, when co-cultured with embryonic neural progenitor cells (NPCs), promote stem cell maintenance *via* unknown soluble factors (Gama Sosa et al., 2007; Vissapragada et al., 2014). Similar co-culture system of ECs with embryonic mouse spinal cord stem cells was also shown to enhance NSC survival and preserve their multipotency (Lowry et al., 2008). An interesting study using neonatal NSCs co-cultured with brain ECs revealed a physical interaction of these cells *via* NSC-expressed integrinα6β1 and EC-expressed laminin (Rosa et al., 2016). This interaction promoted NSC proliferation partly *via* the Notch and mammalian target of rapamycin (mTOR) signaling cascades (Rosa et al., 2016).

Studies in the developing hindbrain have demonstrated that RC2-positive NPC processes physically interact with the germinal zone vasculature (Tata et al., 2016). Compared to the hindbrain, in the neocortex, PVP patterning coincides with the generation of the Tbr2+ BPs and these progenitors closely associate with the incoming PVP (Javaherian and Kriegstein, 2009). Interestingly, in situations of an aberrant vasculature due to ectopic expression of vascular endothelial growth factor (VEGF), Tbr2+ cells remain closely associated and grow in alignment with the developing vasculature (Javaherian and Kriegstein, 2009), thus further highlighting the need of the vasculature for progenitor proliferation. However, the molecular mechanisms delineating their association remain to be elucidated.

The CNS is covered and protected by the meninges comprising of dura-mater, arachnoid and pia-mater. These layers are rich in blood and lymphatic vessels, as well as nerve supply. Interestingly, in contrast to the general concept that neural precursors inhabit the parenchymal tissue, increasing evidence suggests that the meninges also contain multipotent stem cells that possess neurogenic signature and contribute to the CNS formation (Bifari et al., 2009, 2015, 2017; Decimo et al., 2011; Nakagomi et al., 2012; Ninomiya et al., 2013; Kumar et al., 2014). Generated during E13.5–E16.5, these quiescent radial glia-like, nestin-positive stem cells migrate into the neocortex early after birth, and differentiate into functional cortical interneurons and projection neurons (Bifari et al., 2017). Whether meningeal blood and lymphatic vessels regulate the properties of these stem cells remains unknown.

It is worthwhile to mention that oligodendrocyte precursor cells (OPCs), a type of glial cells that give rise to mature oligodendrocytes, also associate with blood vessels during development (Seo et al., 2014; Maki et al., 2015; Tsai et al., 2016). In the presence of extracellular signaling cues, OPCs in culture can be reprogrammed into multipotent CNS stem cells, can self-renew and give rise to oligodendrocytes, astrocytes and neurons (Kondo and Raff, 2000; Gaughwin et al., 2006). It is tempting to speculate that these extracellular cues could be initiated by the local vasculature in response to specific needs of the growing tissue.

Blood vessel-derived extracellular matrix (ECM) is necessary for proper attachment of radial glial endfeet and proper NSC-vessel interaction. A recent report showed that endothelial Dab1 signaling regulates the laminin- and integrin-mediated association of RGCs and astrocytes in the developing brain (Segarra et al., 2018). Loss of endothelial Dab1 decreases the deposition of Laminin-α4, thus causing detachment of radial glial endfeet from the basement membrane. This subsequently leads to defects in the glia-dependent neuronal migration and soma translocation during brain development, thereby, indicating that EC-derived ECM is important for neurodevelopment.

A gene expression study in cultured neonatal NPCs emphasized the differential gene expression of metabolic pathway regulators during different NPC fates (Karsten et al., 2003), implicating dynamic metabolic demands of progenitors during proliferation and cell fate decisions. Interestingly, changes in the gestational metabolism leading to hyperglycemia result in impaired embryonic neocortical RGC differentiation potential and lead to a defective RGC-scaffold (Rash et al., 2018), indicating that energy supply by blood vessels is crucial for proper neurodevelopment. Whether those changes in neural progenitors are regulated by blood vessels-derived factors or differential transport is a possibility that remains unexplored.

### The Role of Hypoxia

The early embryonic brain is hypoxic due to the absence of vasculature. This hypoxic tissue is an ideal niche for harboring proliferative NPCs (Lee et al., 2001; Zhu et al., 2005; Lange et al., 2016). *In vitro* work by Studer et al. (2000) determined the physiological role of oxygen in mesencephalic precursor cell cultures. Low O_2_ concentration (~3%) favored proliferation in these precursor cells and eventually accelerated the total dopaminergic neuron production. Further studies to understand the molecular mechanisms underlying the role of oxygen *in vitro* and *in vivo*, showed that the transcription factor, Hif1α acts as a positive regulator for *in vivo* survival, growth and differentiation of NPCs (Tomita et al., 2003; Milosevic et al., 2007). Hif1α conditional knockout in neural cells resulted in extensive loss of neurons *via* apoptosis, while the neocortex displays hydrocephaly (Tomita et al., 2003). On similar lines, Lange et al. (2016) recently demonstrated that *in vivo* vessel ingression in the neocortex regulates the switch from initial RGC (the apical NPCs during neocortical development) expansion towards a differentiation mode by relief of hypoxia. In a more detailed fashion, the metabolic regulation of stem cells by the vasculature was shown for the first time. Using GPR124 knockout mouse embryos, which display a reduced vascular network specifically in the CNS and thus higher hypoxia, the authors described that stabilization of Hif1α in NPCs leads to an increase in the expression of glycolytic genes such as pfkfb3, accompanied by an increase in RGC expansion. They also showed that Pfkfb3 prevents RGC differentiation and thereby maintains the proliferative niche of the progenitors (Lange et al., 2016). Thus, relief of hypoxia by blood vessels is required for the metabolic switch needed in progenitors to initiate differentiation.

Studies in the developing mouse hindbrain show that the peak in NPC mitosis positively correlates with angiogenic sprouting. Interestingly, Neuropilin1 (NRP1) expressed by ECs was shown to positively regulate the mitotic behavior of the NPCs, as EC-specific deletion of NRP1 resulted in precocious cell cycle exit of NPCs, independent of the tissue oxygenation levels (Tata et al., 2016). These results indicate that tissue oxygenation and hypoxia may not be the only regulatory mechanism *via* which vessels regulate NPCs and suggest that an active angiocrine signaling may also be involved. Figure 1B highlights the known cues derived from the ingressing vasculature during CNS development. Interestingly, recent RNA sequencing of ECs from the developing forebrain at different stages revealed dynamic changes in gene expression including genes that could act as potential angiocrine molecules, as well as genes involved in metabolism (Hupe et al., 2017). Although these metabolic markers may be required for EC metabolism itself, it is tempting to speculate, and needs to be demonstrated, that the metabolic modulations in CNS ECs during development resulting in EC-derived angiocrine factors could influence NPC function.

## The Adult Neurogenic Niches

For many years the generation of new neurons was considered a privilege of embryonic and early postnatal CNS tissues. However, the birth of new neurons and glia takes place also in the adult mammalian brain and continues throughout life, a process known as “adult neurogenesis” (Ming and Song, 2011). NSCs generating neurons and glia have been identified in different locations within the adult brain such as the subgranular zone (SGZ) in the dentate gyrus (DG) of the hippocampus, the SVZ of the lateral ventricles and the hypothalamus adjacent to the third ventricle wall (Kokoeva et al., 2007; Lin and Iacovitti, 2015). Similar to the embryonic stages of CNS development, recent studies have also highlighted the idea of the leptomeninges in adult rat brain and spinal cord as potential niches that harbor stem/precursor cells with a neurogenic potential and that can functionally participate in parenchymal reaction during spinal cord injury (Bifari et al., 2009; Decimo et al., 2011; Nakagomi et al., 2012).

**Figure 2:**
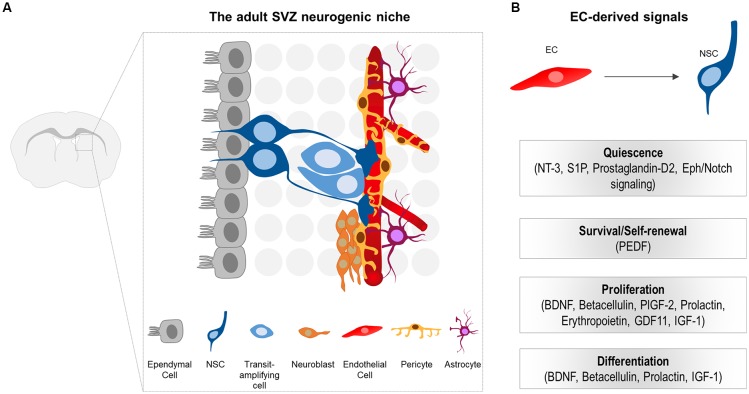
**(A)** Schematic illustration of the subventricular zone (SVZ) neurogenic niche in the adult mouse brain. Neural stem cells (NSCs) generate transit-amplifying cells, which in turn give rise to the migrating neuroblasts. NSCs are located beneath the ependymal cell layer. The basal processes of NSCs contact the ECs of the vessels. Note that pericytes, as well as the endfeet of NSCs and astrocytes tightly enwrap the blood vessels. NSCs processes, pericytes and niche astrocytes form the neurovascular unit (NVU), important for controlling the specific EC blood-brain barrier (BBB) properties at the niche. **(B)** EC-derived cues that promote quiescence, survival, proliferation and differentiation of NSCs and their progeny in the adult CNS.

The SVZ represents the largest germinal zone in the adult brain. NSCs (type B cells) of the SVZ are astrocyte-like cells that exist in a quiescent state (Codega et al., 2014; Mich et al., 2014). Once activated, they give rise to transit-amplifying precursors (type C cells or TACs) which, in turn, generate neuroblasts (type A cells) or glia (Figure 2A; Kriegstein and Alvarez-Buylla, 2009; Ming and Song, 2011). Neuroblasts migrate along the rostral migratory stream (RMS) to the olfactory bulb (OB), where they differentiate into mature interneurons (Kriegstein and Alvarez-Buylla, 2009; Ming and Song, 2011). In contrast to the SVZ, NSCs of the SGZ generate neuroblasts that migrate short distances into the DG and mature into dentate granule neurons (Zhao et al., 2008; Bonaguidi et al., 2012).

In the adult neurogenic niches, NSCs reside in a specialized microenvironment where they interact with a variety of cell types that influence their behavior. Cell components of the adult SVZ niche include NSCs and their progeny, ependymal cells that line the cerebrospinal fluid (CSF)-filled ventricles, neurons, non-stem cell astrocytes, microglia, as well as components of the vasculature (ECs and pericytes; Figure 2A; Ihrie and Alvarez-Buylla, 2011; Bjornsson et al., 2015). Unlike B cells in the SVZ, NSCs in the SGZ differ in location and are rather embedded deeper in the brain parenchyma away from the walls of the ventricle, surrounded by neurons, glia, and blood vessels (Fuentealba et al., 2012).

NSCs of the SVZ lie beneath the ependymal cell layer. They exhibit a polarized morphology reminiscent of their embryonic predecessors, the radial glia. More specifically, they extend a short apical process projecting through the ependymal cell layer to directly access the CSF (Mirzadeh et al., 2008). Additionally, they extend a long basal process that contacts blood vessels through specialized endfeet (Figure 2A; Mirzadeh et al., 2008; Fuentealba et al., 2012). Similar to the apical-basal morphology observed in SVZ NSCs, radial astrocytes of the SGZ (that serve as NSCs) are highly polarized with a proximal domain that faces the hilus and includes contacts with blood vessels, a primary cilium, and lateral processes that contact other radial astrocytes (Fuentealba et al., 2012). The more distal domain is highly branched and contacts neuronal processes and other glial cells (Fuentealba et al., 2012). Therefore, NSCs in both the SVZ and SGZ are poised to receive signals from the vascular compartment.

## Vascular Regulation of Adult Neurogenesis

The perivascular niche for neurogenesis was first described in the adult hippocampus as the anatomical interconnection of dividing ECs with newly generated neurons (Palmer et al., 2000). Since then, a notable number of studies focused on the vital role of the vasculature in stem cell niches and identified an impressive repertoire of both EC-secreted, as well as membrane-bound, signaling molecules involved in stem cell homeostasis (Figure 2B). The main findings of important studies will be described in the paragraphs below.

### Specialized Vasculature in Neurogenic Niches

The vasculature in both the adult SGZ and SVZ is highly organized with unique architecture compared to non-neurogenic brain regions (Shen et al., 2008; Tavazoie et al., 2008; Sun et al., 2015). More specifically, both neurogenic niches are characterized by a dense network of planar, interconnected and relatively non-tortuous (straight) blood vessels that provide a substrate for NSCs and their progeny (Shen et al., 2008; Tavazoie et al., 2008; Culver et al., 2013; Sun et al., 2015). However, even though the vascular beds of the SVZ and SGZ both support adult neurogenesis, the SVZ vasculature appears to have distinct features (Tavazoie et al., 2008). In contrast to other areas of the brain, where the integrity of the blood-brain barrier (BBB) is strictly maintained by EC tight and adherens junctions, pericyte coverage and astrocytic endfeet, a modified BBB with specialized features has been proposed to exist in the SVZ (Tavazoie et al., 2008). Intriguingly, small tracer molecule studies have shown that the SVZ has a partially permeable BBB that allows the access of signals deriving from the blood (Tavazoie et al., 2008). These blood-derived signals influence the behavior of NSCs and regulate fate specification, differentiation, quiescence and proliferation. Furthermore, NSCs and their immediate progeny (C cells) directly contact ECs with their basal processes and their cell bodies, respectively, at specialized sites that lack complete coverage by pericytes and astrocytic endfeet, suggesting that a direct NSC-EC communication might also be important for regulating NSC behavior (Tavazoie et al., 2008).

### Effect of the Vasculature in Adult NSCs

#### Endothelial Cell-Secreted Factors

A significant amount of studies show how ECs regulate NSC behavior through secreted factors. BDNF was the first EC-secreted molecule demonstrated to increase neurogenesis—the number of newly generated functional neurons—in the adult songbird brain (Louissaint et al., 2002). *In vitro*, EC-derived BDNF was reported to support neurite outgrowth, survival and migration of newly generated neuroblasts in SVZ explants (Leventhal et al., 1999). Since then, the field gained further insight from *in vitro* studies using NSC/EC trans-well cultures, in which soluble factors released from ECs stimulate self-renewal, inhibit differentiation and enhance neurogenesis of NSCs (Shen et al., 2004). Pigment epithelium-derived factor (PEDF) released by both endothelial and ependymal cells was the first soluble factor shown to selectively increase self-renewal of B cells in the SVZ, and subsequently enhance neurogenesis, *via* potentiating Notch-dependent transcription (Ramírez-Castillejo et al., 2006; Andreu-Agulló et al., 2009). Similarly, betacellulin (BTC), a member of the EGF family expressed by ECs of capillaries and by the choroid plexus, induces expansion of NSCs and neuroblasts by acting *via* both the EGF and ErbB4 receptors located on NSCs and neuroblasts, respectively (Gómez-Gaviro et al., 2012). The chemokine stromal-derived factor (SDF1; also called CXCL12), expressed by the vascular plexus as well as by ependymal cells, was also shown to have a different effect on different stages of the NSC lineage *via* binding to the CXCR4 receptor and thus promoting homing of active NSCs (aNSCs) and TACs to blood vessels (Kokovay et al., 2010). A very recent study has further demonstrated that expression of SDF1 is specifically restricted in capillaries, and that aNSCs and their progeny are preferentially associated with these. In contrast, qNSCs are most prevalent near SDF1-negative vessels (Zhu et al., 2019). An *in vitro* study has identified the placental growth factor 2 (PlGF-2), a ligand of VEGF receptor 1 (VEGFR1), as an EC-derived factor that can promote proliferation of SVZ stem cells and their progeny (Crouch et al., 2015). More recent studies have also shown that diffusible signals enforce quiescence and promote stem cell identity. More specifically, ECs secrete neurotrophin 3 (NT-3) to support the quiescence of NSCs that express the tropomyosin-related kinase C (TrkC) receptor (Delgado et al., 2014). In addition, sphingosine-1-phosphate (S1P) and prostaglandin-D_2_, two EC-derived GPCR ligands, have been shown to actively maintain quiescence of NSCs (Codega et al., 2014). Altogether, these studies demonstrate that EC-derived factors can simultaneously enforce quiescence and promote proliferation depending on the activation state of NSCs, thus suggesting a dual regulation along the lineage.

#### Cell-Cell Interactions

As indicated above, NSCs are in direct contact with blood vessels through their long basal process and their specialized endfeet. A few studies have investigated the importance of these direct cell-cell interactions between ECs and NSCs and showed how they enforce quiescence and promote stem cell identity. More specifically, ECs express ephrinB2 and Jagged1 in their membrane and through activation of Eph and Notch signaling, respectively, they promote quiescence in NSCs that contact them through their basal process (Ottone et al., 2014). In addition to this study, the integrin-mediated signaling was also shown to play a functional role in binding SVZ stem cells within their niche. More specifically, adult NSCs express the laminin receptor α6β1 integrin, thus enabling binding of these cells to the laminin-rich environment around blood vessels. This α6β1 integrin signaling is important for NSCs to bind to ECs, as blocking α6β1 *in vivo* leads to SVZ progenitor cells migration away from the vasculature (Shen et al., 2008).

#### Circulating Effectors

Blood circulating substances could access the SVZ neurogenic niche either directly *via* the partially permeable BBB of the SVZ (based on the results from Tavazoie et al., 2008) or indirectly *via* the choroid plexus/CSF and have been shown to affect the neurogenic niche. For example, prolactin, a hormone upregulated during pregnancy and carried by the bloodstream, has a crucial contribution in enhancing neurogenesis in the SVZ during pregnancy (Shingo et al., 2003). Moreover, circulating erythropoietin can cross the BBB as an intact molecule and serve as an enhancing stimulus for stem cell progenitors during embryonic development, as well as a paracrine neuroprotective mediator of ischemia in the brain (Ruscher et al., 2002). An exciting demonstration of blood-borne circulating factors stimulating SVZ neurogenesis was shown using heterochronic parabiosis models, i.e., linking the circulations of young and old mice. In this study, GDF11 was identified as a factor necessary for increasing neurogenesis in old mice (Katsimpardi et al., 2014). Circulating factors may also have a negative impact on neurogenesis. For example, both corticosterone and the chemokine CCL11 were shown to inhibit neurogenesis (Villeda et al., 2011).

As mentioned above, the vasculature plays an important role in orchestrating NSC fate in the adult CNS during homeostasis. However, its instructive role goes beyond physiological conditions and it appears to be crucial during and after pathological insults, such as cerebral infarction. More specifically, a number of studies demonstrated that ECs promote survival, proliferation and neuronal differentiation of neural stem/progenitor cells in the post-stroke cortex (Nakagomi et al., 2009; Nakano-Doi et al., 2010). After stroke, blood vessels in the injured region upregulate SDF1 and angiopoietin 1 (Ang1) expression, thus attracting neuroblasts into the peri-infarct area and promoting neurogenesis and functional recovery (Ohab et al., 2006). Similarly, in the ischemic striatum, ECs synthesize BDNF, thus promoting recruitment and vasculature-mediated migration of neuroblasts to the injury site (Grade et al., 2013). These studies highlight an additional role of blood vessels as scaffold for migrating neuroblasts into infarcted areas (Kojima et al., 2010; Grade et al., 2013). However, this topic is out of our scope for this review and refer the reader to Saghatelyan (2009); Ding et al. (2013); Sawada et al. (2014); Ruan et al. (2015) and Horgusluoglu et al. (2017) for further details and information.

## Metabolism and Energy-Sensing Mechanisms in Adult NSCs

Adult NSCs constitute a very dynamic population and recent NSC transcriptome analyses have revealed that the transition along the neurogenic lineage is coupled with changes in their metabolic characteristics (Llorens-Bobadilla et al., 2015; Shin et al., 2015). More specifically, in their quiescent, and therefore hypometabolic state, NSCs preferentially utilize glycolysis and fatty acid oxidation (FAO; lipolysis) to support their energy needs (Ito and Suda, 2014; Llorens-Bobadilla et al., 2015; Shin et al., 2015; Stoll et al., 2015; Xie et al., 2016; Knobloch and Jessberger, 2017; Knobloch et al., 2017). Conversely, in highly proliferative aNSCs and their differentiated progeny mitochondrial oxidative phosphorylation (OXPHOS) as well as *de novo* lipogenesis take over to support cell division (Knobloch et al., 2013; Ito and Suda, 2014; Llorens-Bobadilla et al., 2015; Shin et al., 2015). Similarly, NSCs of the embryonic brain exhibit high glycolytic activity necessary for their expansion and/or maintenance, which is reduced upon their differentiation (Lange et al., 2016). These studies imply that metabolic input and nutrient availability are crucial modulators of neurogenesis and contribute to NSC decisions. The vasculature, therefore, emerges as a key regulator of NSC metabolism as it supplies the brain with nutrients and oxygen and ensures that the energy demands of NSCs are met.

### Oxygen Availability and HIF Signaling

Similar to their embryonic predecessors, adult NSCs reside in niches characterized by low oxygen levels (<1%–6%), thus emphasizing its importance in stem cell function (Ochocki and Simon, 2013). Numerous *in vitro* studies have investigated the role of oxygen in NSC self-renewal and fate specification, and demonstrated that low oxygen levels are beneficial for NSCs as they promote their proliferation and survival. NSCs respond to hypoxia by shutting down OXPHOS in favor of glycolytic metabolism. This is orchestrated by the hypoxia-inducible transcription factors (HIFs), which are stabilized and activated under low oxygen availability (<9%; Majmundar et al., 2010). HIF1α signaling is essential to normal NSC function. For example, specific deletion of Hif1α in NSCs of adult mice leads to significant decrease of NSCs in the adult SVZ, thus highlighting the importance of oxygen and oxygen-sensing mechanisms in regulating self-renewal, proliferation and differentiation of NSCs both *in vitro* and *in vivo* (Li et al., 2014). Interestingly, NSC-encoded Hif1α is also important for maintaining vascular integrity in the adult SVZ, as well as for stabilizing the vasculature following brain injury (Roitbak et al., 2008; Li et al., 2014). Furthermore, hypoxia has been linked to Wnt/β-catenin signaling in NSCs by showing that oxygen availability has a direct role in NSC regulation through Hif1α modulation of Wnt/β-catenin signaling (Mazumdar et al., 2010). Despite those initial findings, the underlying molecular mechanisms of Hif1α action in adult tissue remain elusive.

In addition to oxygen, nutrients comprise an important regulator of adult neurogenesis. As indicated in the previous paragraphs, nutrients, growth factors and circulating hormones, can be delivered by the vasculature either by diffusion or through a transport-mediated system and influence the behavior of NSCs. This implies that there are molecular mechanisms that respond to nutrient availability and orchestrate NSC response to energy alterations (e.g., caloric restriction, exercise, pathologies, aging, etc.). Among these mechanisms sirtuins, CREB, AMPK, and the insulin/IGF pathway are the best-characterized ones. In this review, we will focus on the latter as it comprises a central regulator for the development and function of the CNS through the activation of numerous downstream signaling cascades. For further details on the other pathways, we refer the reader to the recent publications (Cantó and Auwerx, 2010; Rafalski and Brunet, 2011; Houtkooper et al., 2012; Ochocki and Simon, 2013; Ito and Suda, 2014; Fusco et al., 2016).

### The Insulin/IGF Signaling Pathway

One of the brain’s mechanisms to respond to glucose and energy excess is the insulin/IGF-1 signaling pathway. Systemic IGF-1 and insulin can both cross the BBB and bind to their tyrosine kinase receptors leading to their auto-phosphorylation (Hubbard, 2013; Kavran et al., 2014). Recently, a study focusing on neurovascular coupling has demonstrated that neuronal activity can induce changes in BBB permeability thus promoting the release and entrance of IGF-1 into the CNS, and consequently leading to an increase in its availability (Nishijima et al., 2010). The receptors for insulin/IGF-1 are highly expressed in NSCs in neurogenic niches, and several studies have implicated insulin/IGF-1 signaling in NSC maintenance, proliferation and differentiation (Rafalski and Brunet, 2011). More specifically, *in vivo* infusion of IGF-1 induces NSC proliferation and subsequent neurogenesis in the adult rat hippocampus (Aberg et al., 2000). Similarly, IGF-1 has a direct proliferative effect in adult hippocampal NSCs *in vitro* (Aberg et al., 2003). Even though the role of insulin in adult NSCs *in vivo* has not been elucidated, *in vitro* studies have demonstrated that insulin can induce neurogenesis (Han et al., 2008; Yu et al., 2008; Rhee et al., 2013). The main mediator of insulin/IGF-1 signaling in NSCs is the PI3K/Akt signal transduction pathway and many downstream signaling components have been shown to be involved in NSC biology, including FoxO transcription factors and mTOR (Rafalski and Brunet, 2011).

#### FoxO Transcription Factors in NSCs

FoxO transcription factors have been shown to be essential for both embryonic and adult stem cells (Rafalski and Brunet, 2011; Rafalski et al., 2012). Gene expression analysis in adult NSCs shows that FoxO transcription factors, and in particular FoxO3, induce a specific program of genes that preserves quiescence, and controls glucose and oxygen metabolism thus highlighting their role in NSC homeostasis (Renault et al., 2009). Accordingly, in the absence of FoxOs NSCs hyperproliferate, leading to the exhaustion of the quiescent stem cell pool (Renault et al., 2009). FoxOs are negatively regulated by the insulin/IGF-1 pathway through the PI3K/Akt branch, thus suggesting a direct link between nutrient availability and stem cell metabolism (Rafalski and Brunet, 2011).

#### mTOR Signaling in NSCs

The mTOR is a central regulator of cell homeostasis and protein synthesis. In neurogenic niches, several studies have highlighted its role in many aspects of neurogenesis as it is involved in fine-tuning the balance between stem cell self-renewal and differentiation (Magri and Galli, 2013; LiCausi and Hartman, 2018). For example, recent *in vivo* studies in adult mice have demonstrated that mTOR activation promotes NSC proliferation and subsequent neuronal differentiation, at the expense of quiescence and self-renewal (Paliouras et al., 2012). In contrast, sustained mTOR activation in embryonic NSCs leads to premature differentiation and apoptosis at the expense of the stem cell pool (Magri et al., 2011; Kassai et al., 2014). mTOR can be activated in response to insulin/IGF, nutrients such as glucose and amino acids, as well as pro-inflammatory cytokines (e.g., TNFα, CD95; Magri and Galli, 2013; LiCausi and Hartman, 2018). In contrast, many cellular stresses such as hypoxia and low energy act to inactivate mTOR (Magri and Galli, 2013; LiCausi and Hartman, 2018).

## Conclusions and Perspectives

In the past years, significant progress has been made to support the concept of a perivascular niche that regulates stem cells, and blood vessels have emerged as an integral component of both embryonic and adult neurogenic niches. In the developing brain, current knowledge on how blood vessels regulate NSCs is limited, in part due to the limitations of working with mouse embryos. Emerging new technical approaches, such as whole tissue imaging and single cell sequencing, will rapidly pave the path towards a better understanding of cell-cell interactions and molecular signaling pathways required for proper development of the CNS, and in particular towards the vascular control of NSC properties. Accruing to this, recent sequencing data obtained from embryonic mouse CNS tissue describes an interesting list of genes expressed by ECs during development, which could act as angiocrine factors and directly regulate NPC properties (Lange et al., 2016; Hupe et al., 2017). However, their influence on the NPCs still needs to be addressed.

Similarly, in the adult SVZ and SGZ, the close interaction of blood vessels and NSCs has a substantial impact on the behavior of the latter. An important number of studies have demonstrated that EC-derived factors, as well as direct NSC-EC interactions, can affect NSC self-renewal, proliferation, differentiation, and survival. It is now recognized that this NSC lineage progression from quiescence to activation is characterized by alterations in their metabolic status. However, whether and how signals derived from the vasculature, or how physiological remodeling of the vasculature, are directly “translated” into the metabolic switches that accompany the cellular states of NSCs needs to be further explored. In this respect, nutrients are necessary for neurogenesis, and NSCs have developed a repertoire of sensing mechanisms to respond to nutrient availability. As blood vessels comprise the main conduits for nutrients and oxygen, it would be of great interest to investigate whether NSCs can “talk” back to ECs to regulate nutrient availability for their own demands.

## Author Contributions

All the authors listed contributed to the concept and design of the manuscript. AK and BS wrote the manuscript and prepared the figures. CRA wrote and critically revised the manuscript.

## Conflict of Interest Statement

The authors declare that the research was conducted in the absence of any commercial or financial relationships that could be construed as a potential conflict of interest.
